# Long-term results after the one-stage posterior-only surgical correction of thoraco-lumbar kyphoscoliosis in congenital spine deformity caused by two ipsilateral hemi-vertebrae

**DOI:** 10.1186/s12891-021-04201-z

**Published:** 2021-04-02

**Authors:** Xuhong Xue, Sheng Zhao, Feng Miao, Kai Li, Bin Zhao

**Affiliations:** Department of Orthopedics, The Second Hospital of Shanxi Medical University, No. 382 Wuyi Road, Taiyuan, Shanxi 030001 People’s Republic of China

**Keywords:** Congenital kyphoscoliosis, Two ipsilateral hemivertebrae, Thoracolumbar, Hemivertebra resection, Curve progression

## Abstract

**Background:**

Two ipsilateral hemivertebrae is less common and presents severe growth imbalance caused by the vertebral anomalies. However, there is a paucity of reports regarding to two ipsilateral thoracolumbar hemivertebrae. The purpose of present study is to evaluate the long-term outcomes of the posterior surgical correction of thoraco-lumbar spine deformity caused by two ipsilateral hemivertebrae.

**Methods:**

From 2006 to 2014, a total of 14 consecutive pediatric patients with congenital thoraco-lumbar hemivertebrae were treated by posterior excision of hemivertebrae with short segment fusion. The following parameters were measured: coronal major curvature, cranial and caudal compensatory curvature, segmental kyphosis, lumbar lordosis, trunk shift, apical vertebra translation and sagittal vertical axis. These results were compared and evaluated in preoperatively, immediately postoperatively and at the final follow-up. All patients had a minimum of 5 years follow-up.

**Results:**

The mean age at surgery was 11.1 ± 4.8 years (2yos to 17yos). The mean follow-up period was 80.2 ± 19.4 months (60mons to 117mons). There was a mean improvement of 74.2% in the coronal major curve from a mean angle of 64.1° before surgery to 15.8° at the final follow-up. The cranial and caudal curves improved of 69.8 and 69.0% from 25.6° to 7.7°, 26.9 to 8.2, respectively. The mean thoraco-lumbar kyphosis was 59.9° before and 13.6° after surgery, 20.8° at the final follow-up. Alignment in the coronal and sagittal plane was either maintained or improved within normal values in all patients.

**Conclusions:**

Good correction and spinal balance can be achieved by posterior-only hemivertebrectomy in patients with thoracolumbar kyphocsoliosis caused by two ipsilateral hemivertebra. The complication of neurological injury is low but a technically demanding procedure. More attention should be paid in residual curve progression after surgery.

## Background

The natural history of congenital scoliosis has been well documented [1, 2]. The type and location of the vertebra deformity determines the severity and prognosis of the congenital spinal abnormalities. Hemivertebra (HV) is the most common cause of congenital scoliosis; especially when the HV is fully segmented or semi-segmented, progression of curve is usually unavoidable [3]. According to the report of Bollini G, thoracolumbar HV have a singular behavior; the deteriorating rate of the scoliosis or kyphosis is more severe in the thoracolumbar region [4]. Main driving force for the scoliosis is the severe growth imbalance produced by the hemivertebrae.

Two ipsilateral HV are not common but have a much worse prognosis. The hemivertebrae are usually separated by normal vertebrae. Full segmented HV means the absence of four growth plates on concave side of the curvature, resulting in much greater growth imbalance. Prior study suggested that kyphoscoliosis due to anterolateral unsegmented bar combined with posterolateral quadrant HV progress at 5° per year until the age of 10 and 10° thereafter [5]. McMaster et al. had reported the natural history of thoracolumbar HV in patients younger than 5 years old. They found that the main curve progression varied from 41° to 148° during a mean 4-years follow-up [1]. Most importantly, most of the patients had progressive kyphosis in thoracolumbar junction, which always lead to neurological deficits [6]. Therefore, all of them require surgical treatment to achieve symmetric growth of the spine at the early stage, avoiding more serious spinal deformities.

There are various types of surgical procedures in the treatment of congenital kyphoscoliosis. Combined anterior and posterior convex hemiepiphysiodesis, excision of HV via posterior or anterior-posterior fusion techniques have been used extensively [7, 8]. However, the surgical treatment for severe spinal deformities in children is extremely challenging. In-situ fusion or hemiepiphysiodesis at smaller ages can slow down or arrest the growth, achieving limited correction rates [9]. The definitive treatment of the congenital kyphoscoliosis caused by HV should include removal of HV. Recently, HV excision via posterior approach or single level apical osteotomy in pediatrics has been reported as the choice of treatment with successful results [10–12].

There is a paucity of report regarding to congenital kyphoscoliosis caused by two ipsilateral HV. The aim of this study is to evaluate the long-term outcomes of the one-stage posterior-only surgical correction of thoraco-lumbar kyphoscoliosis caused by two ipsilateral HV.

## Methods

This retrospective study was approved by the Ethics Committee of our Hospital. From 2006 to 2014, we identified all patients with the diagnosis of congenital thoracolumbar kyphoscoliosis, among of these patients who had two ipsilateral HV were reviewed from our database. The thoracolumbar region was defined as range from T10 to L2. Of all, fourteen consecutive patients were included with a follow-up of at least 5 years.

Demographic data were recorded including sex, age, height, body weight and Risser sign. In addition to the radiological data, inpatient and outpatient records were reviewed. Preoperative evaluation included a neurologic examination, full length radiographs in anterioposterior and lateral view. 3-D computer tomography in entire spine was performed to detect details of spine deformity, and MRI is for detecting intraspinal abnormalities and spinal cord malformation. To detect congenital heart and urogenital abnormalities, Cardiovascular and urogenital ultrasounds were also performed. No patient had undergone a prior surgery. No patient had a neurologic deficit.

All patients underwent posterior hemivertebra resection in primary surgery. For patients needed revision surgery, PSO or Y-Shape osteotomy was performed depending on the status of spine deformity. The fusion level depends on severity of spine deformity, as well as scope and flexibility of curve. For very young children, one vertebra above and below after HV resection were fixated and fused. If the scoliotic curve was large or with focal kyphosis, two vertebrae above and below after HV resection were fused. Of course, for severe and rigid scoliosis or kyphoscoliosis, more fusion level may be needed. All radiographs were measured by two spine surgeons independently based on free standing posteroanterior and lateral X-ray taken in preoperatively, immediately after surgery and at the final follow-up. The location and number of the HV, relationship of the adjacent vertebrae (segmented status), coronal major curve, cranial and caudal compensatory curve, apex vertebra translation, trunk shift, segmental kyphosis, lumbar lordosis, thoracic kyphosis and SVA were record. The segmental scoliotic and kyphotic curves were measured according to Cobb’s method. The trunk shift (TS) was evaluated as the distance between a vertical line drawn from the center of C7 vertebral body to the middle of sacrum, which was related to the pelvis width (the distance between the two points of the iliac crests tangential to the bi-iliac line) and expressed in percentage to avoid effects of radiographic enlargement [13]. These parameters were compared and evaluated in preoperatively, immediately postoperatively and at the final follow-up.

After surgery, all patients were required to a rigid brace for at least 3 months, aiming to protect the instrumentation. All patients were followed up at 1 week, 1 month, 3 month, 6 month, 1 year at postoperatively, and then every year. The method of fusion evaluation is bending film of X-ray and CT scan.

### Statistics

Pair t-test were used to analyze the difference of coronal major curve, cranial and caudal compensatory curve, segmental kyphosis, lumbar lordosis, apex vertebra translation, trunk shift and SVA at pre-operation, post-operation and final follow-up. The differences with a *P* value less than 0.05 were considered as statistically significant.

## Results

**Fig. 1:**
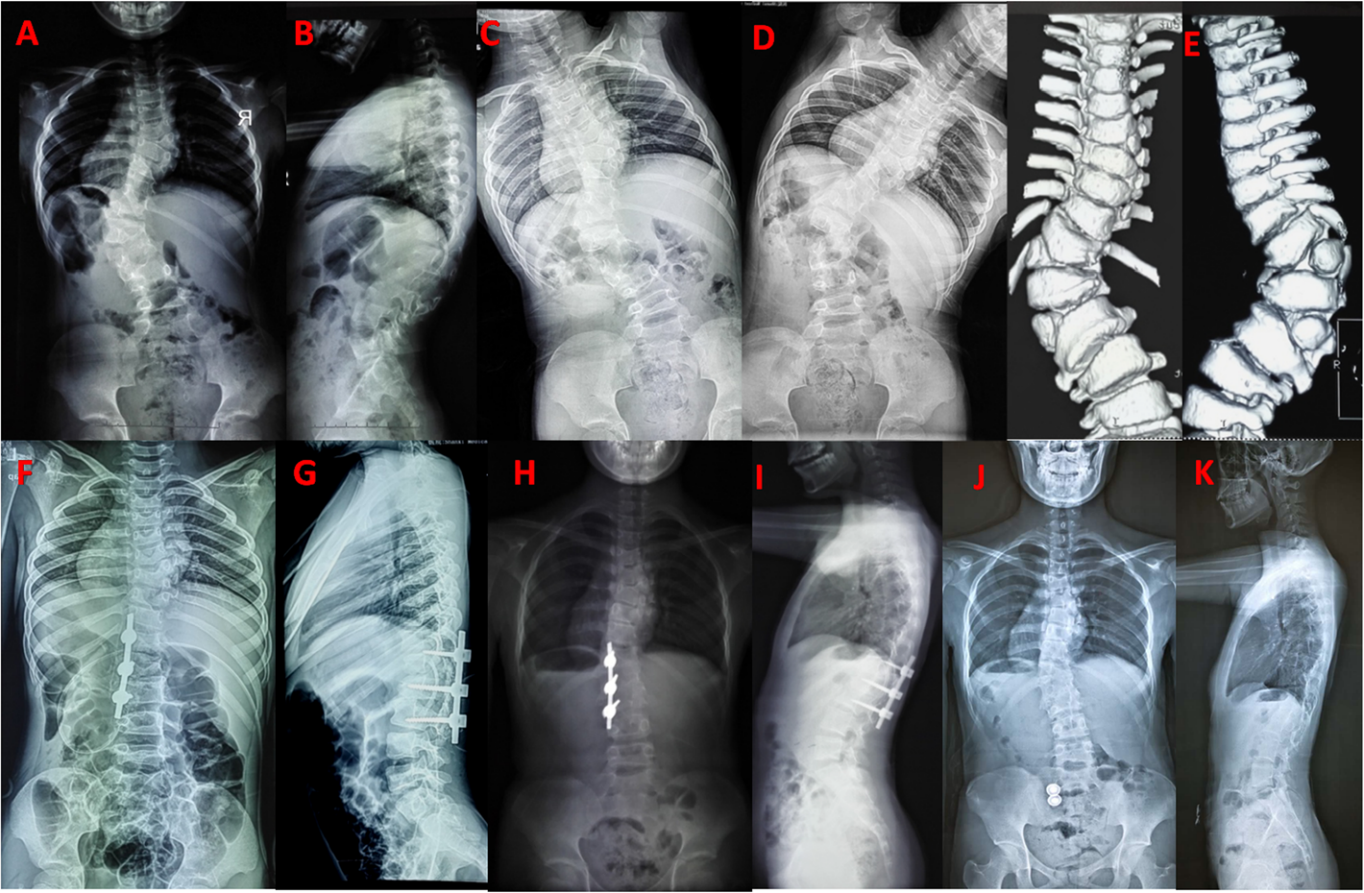
A 8 years old girl with kyphoscoliosis due to T6, 11 and L1 HV. Radiographs images and CT images were obtained preoperatively, which shows T11 and L1 full segmentation HV caused kyphoscoliosis (**a, b, e**), less flexibility and very rigid curve (**c,d**). Postoperative PA and lateral radiograph, she was treated with posterior L11, L1 HV resection with unilateral fusion (**f,g**). PA and lateral radiograph at 46 months follow-up show that correction and trunk balance are well maintained (**h,i**). Implants removal was required in 6 years after surgery. Final PA and lateral radiograph at 84 months follow-up show that residual curve and trunk balance are stable (**j,k**)

Of the all patients, there are seven girls and seven boys. The mean age at surgery was 11.1 ± 4.8 (3 to 17) years. The mean follow-up period was 80.2 ± 19.4 (60 to 117) months. One stage posterior hemivertebra resection with unilateral short fusion in patients less than 10 years old was performed (Fig. 1). For patients more than 10 years old, posterior hemivertebrectomy with bilateral instrumentation and fusion were performed. There are four patients undergoing unilateral fusion and 10 patients undergoing bilateral fusion. The mean fusion level was 5.2(3 to 8) segments. Of all, there were 6 full segmented and 8 semi-segmented HV, 4 cases with concave unsegmented bar. Three cases associated with HV of other sites, 2 cases with bloc vertebra, and 3 cases with rib anomalies. The demographic data and surgical details could be seen in Table 1.

**Table 1:** Demographic data, deformity characteristics and surgery details in two ipsilateral hemivertebrae in thracolumbar region

Cases	Sex	Age	HV location	Side	Segmentation	Associated congenital deformity	Fixation type	ACR	Operative	Fusion level	Follow-up (month)
1	F	3	T10,12	L	FS	10-11th fused rib	Unilateral	No	T10,12 HVR	T10-L2	60
2	F	4	T10,12	R	SS	9th bifid rib	Bilateral	No	T12 HVR,T10 partial resection	T8-L2	69
3	M	8	T10,11	L	SS	*FS*:T10–12; SCM; 10-11th fused rib	Unilateral	No	T10,11 HVR	T7-L1	95
4	F	8	T6,11,L1	R	FS	–	Unilateral	No	T11,L1 HVR	T10-L2	84
5	F	7	T11,Ll,L4	R	FS	–	Unilateral	No	T11.L1 HVR	T10-L2	67
6	M	9	T11,12	L	FS	–	Bilateral	Yes	T12 HVR	T10-L2	61
7	M	11	L1/2,L2	L	SS	T1–3 vertebra bloc; *FS*:L1–3	Bilateral	Yes	L1/2 HVR L2 partial resection	T12-L4	103
8	M	13	T11,L1/2	L	FS	–	Bilateral	No	T11, L1/2 HVR	T11-L4	69
9	M	14	T11,12	L	SS	*FS*:T10–12	Bilateral	No	T11 HVR	T7-L3	103
10	F	15	T8/9,10,L1	L	SS	*FS*:T8-L2 Klipple-Feil syndrome	Bilateral	No	T8/9,10,L1HVR	T8-L5	61
11	F	15	T12,L1	R	SS	*FS*:T11-L2	Bilateral	Yes	T12 HVR	T9-L3	117
12	M	17	T11,12	L	SS	*FS*:T11-L1	Bilateral	Yes	T11,12 HVR	T7-L4	68
13	F	17	T12,L1	R	FS	*–*	Bilateral	Yes	L1 HVR; T12 partial resection	T10-L4	99
14	M	15	T11,12	R	SS	*FS*:T10-L1; L3–4 vertebra bloc; Goldenhar syndrome	Bilateral	Yes	T11,12 HVR	T8-L3	67

Notes: *HV* hemivertebra, *R* hemivertebra located on right side, *L* hemivertebra located on left side, *FS* full segmented, *SS* semi-segmented, *M* male, *F* female, *SCM* spinal cord malformation, *FS* failure of segmentation, *HVR* hemivertebra resection

### General correction results

The mean coronal major curve was 64.1 ± 16.8° (40 to 93) before operation, which improved to 17.1 ± 12.6° (4 to 45), 15.8 ± 11.0° (5 to 42) postoperatively and at the final follow-up, with a mean correction of 73.3 and 75.4%. The mean cranial compensatory curve was 25.6 ± 11.4° preoperatively and 7.7 ± 5.3° at the final follow-up, giving a correction of 69.9%. The mean caudal curve was 26.9 ± 9.3° preoperatively and 8.2 ± 6.1° at the final follow-up, giving a correction of 69.5%. The trunk shift was not significant difference between preoperatively and postoperatively, but had significant difference at the final follow-up. The apex vertebra translation (AVT) was improved significantly before and after operation, and kept stable during the follow-up period (Table 2).

**Table 2:** Radiographic data of patients in coronal and sagittal plane

	Preoperative	Postoperative	Improvement (%)	*P* value	Final follow-up	Improvement (%)	*P* value
Coronal plane							
Segmental curve(°)	64.1 ± 16.8	17.1 ± 12.6	75.5	*0.000	15.8 ± 11.0	74.2	*0.000
Cranial curve(°)	25.6 ± 11.4	8.0 ± 6.0	69.2	*0.000	7.7 ± 5.3	69.8	*0.000
Caudal curve(°)	26.9 ± 9.3	6.9 ± 6.3	73.7	*0.000	8.2 ± 6.1	69.0	*0.000
TS(%)^a^	13.8 ± 12.9	9.4 ± 11.5	31.9	0.295	4.8 ± 5.6	65.2	*0.019
AVT(%)^a^	34.5 ± 13.1	10.5 ± 7.3	69.6	*0.000	12.3 ± 5.8	64.3	*0.000
Sagittal plane							
Segmental TL kyphosis(°)	59.9 ± 23.2	13.6 ± 9.5	77.3	*0.000	20.8 ± 13.8	65.3	*0.000
Thoracic kyphosis(°)	60.6 ± 15.7	34.6 ± 11.0	42.9	*0.001	42.8 ± 12.5	29.4	*0.001
Lumbar lordosis(°)	65.3 ± 13.1	46.6 ± 9.1	28.6	*0.000	45.8 ± 7.6	29.9	*0.000
SVA (mm)	0.85 ± 1.14	1.0 ± 0.9	–	0.692	0.8 ± 0.9	6.2	0.782

Notes: *TS* trunk shift, *AVT* apex vertebra translation, *TL* thoracolumbar, *SVA* sagittal vertebral axis

^a^Trunk shift and AVT were related to the pelvis width and expressed in percentage to avoid effects of radiographic enlargement

*Means there is statistical significant difference

The mean segmental kyphotic curve was 59.9 ± 23.2° (42.6 to 100) before and 13.6 ± 9.5° (0 to 30) after surgery, 20.8 ± 13.8° (2 to 46) at the final follow-up. The mean thoracic kyphosis was 60.6 ± 15.7° (28 to 98) before surgery and 42.8 ± 12.5° (23 to 63) at the final follow-up. The mean lumbar lordosis was 65.3 ± 13.1° (46.8 to 96) before surgery and 45.8 ± 7.6° (31 to 59) at the final follow-up. Spinal alignment in the sagittal plane was either maintained or improved in all patients (Table 2).

### Complications

**Fig. 2:**
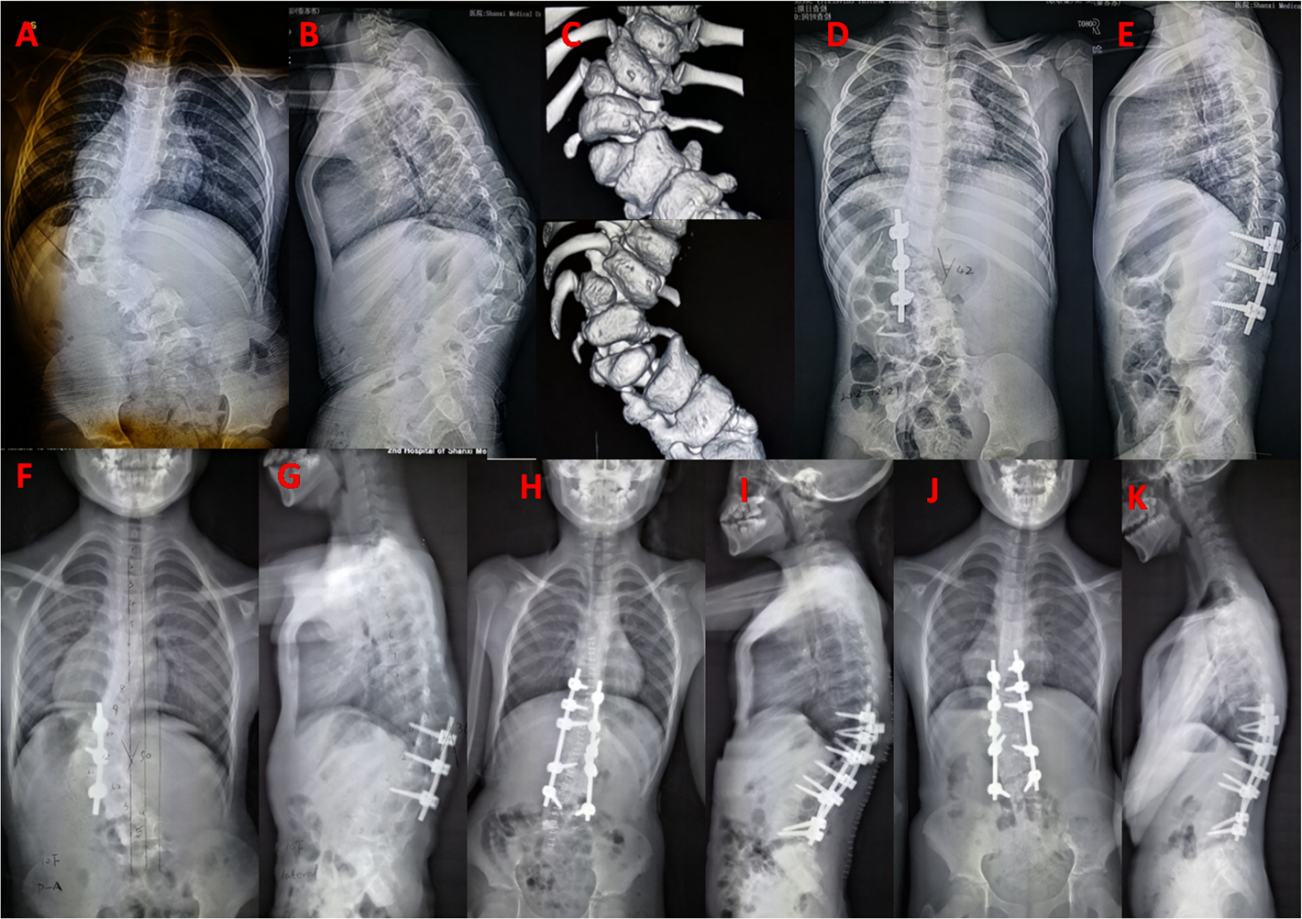
A 7 years old girl with T11, L1 and L4 HV. Radiographs images and 3D-computed tomography images were obtained preoperatively, which shows thoracolumbar kyphoscoliosis due to T11 and L1 full segmentation HV, L4 Semi-segmentation HV (**a, b, c**). Postoperative PA and lateral radiograph, she was treated with posterior L11,L1 HV excisions with convex fusion (**d,e**). Curve progression, PJK and L2 pedicle screw plow were presented in 38 months after surgery (**f,g**). Revision surgery including further hemivertebretomy and extended fixation was performed (**h,i**). Final PA and lateral radiograph at 67 months follow-up show that correction and trunk balance are well maintained (**j,k**)

One patient presented incomplete neurologic injury in hemivetebra resection procedure. She had left lower extremity weakness 4/5 and hyperalgesia, which made a recovery completely after 2 weeks. Rod breakage was found in one patient at 1 year after surgery. She was suggested to observation considering no any discomfort. Three patients required additional surgery during follow-up. The reasons and details of additional surgery were summarized in Table 3. These patients were larger and rigid curve in coronal and sagittal plane before the primary surgery. The revision surgeries including PSO or Y-Shape osteotomy were performed and satisfactory outcomes were achieved at the final follow-up (Fig. 2). All patients achieved solid fusion at the latest follow-up.

**Table 3:** Details of the three patients with two ipsilateral thoracolumbar hemivertebra in revision surgery

Case	Age/Sex	Abnormality	Segment scoliosis (1st pre-,post-OP 2nd pre-,post-OP at final follow up)	Segment kyphosis (1st pre-,post-OP 2nd pre-,post-OP at final follow up)	Revision reason	Initial surgery	Duration time (mo)	Revision surgery	Final F/U (mo)
1	4/F	T10,12 HV (FS)	91.0°-36.0° 51.4°-30.0° 27.4°	50.0°-3.0° 7.1°-10.0° 4.0°	Residual scoliosis progression in proximal region; incomplete resection of T10 HV	T10,12 HV resection T8-L2 convex fusion	33	T10 radical resection with T8-L2 fusion	69
2	7/F	T11,L1 HV (FS); L4 HV (SS)	93.3°-41.0° 57.3°-13.7° 15.3°	77.6°-13.0° 36.0°-18.0° 20.2°	Residual scoliosis progression, PJK and L2 pedicle screw plowed; too short fusion level and T11 incomplete resection	T11,L1 HV resection T10-L2 convex fusion	38	L2 Y-Shape osteotomy with T8-L5 fusion	67
3	13/M	T11 BF and wedge vertebra L1/2 HV (FS)	73.6°-17.9° 43.2°-17.6° 15.7°	41.8°-8.5° 55.4°-12.7° 13.1°	Residual scoliosis progression and PJK;T11 asymmetric growth	L1/2 HV resection T11-L4 fusion	28	T11 PSO with T8-L5 fusion	69

Notes: *F* female, *M* male, *HV* hemivertebra, *BF* butterfly vertebra, *FS* full segementation, *SS* semi-segmentation, *PJK* proximal junction kyphosis

## Discussion

Previous study has reported that kyphoscoliosis in the thoracolumbar junction often have bad prognosis if no proper intervention was performed [14]. For patients with posterior-lateral two ipsilateral thoracolumbar HV, although this condition is less common, paraplegia is a far greater risk. Besides progressive kyphosis, these vertebral abnormalities may also lead to lateral curvature and resulted in kyphoscoliosis. Other than the risk of spinal cord compression in apex vertebra site, it produces a compensatory lumbar hyperlordosis causing low back pain. Therefore, surgical treatment usually was required at the early age to prevent further severe spinal deformity.

In spite of the developments by leaps and bounds in spinal surgical technologies in three decades, the treatment of congenital scoliosis caused by HV is still controversial. Excision of the HV addresses the deformity directly and allows reliable correction immediately in very young patients. Satisfactory correction and restoration of balance can be achieved [15]. Generally speaking, severe global imbalance is not common if curve located in the thoracolumbar spine. Because of the compensatory space was enough in coronal plane (cranial and caudal) and sagittal plane (thoracic kyphosis and lumbar lordosis). In present study, posterior hemivertebrectomy with unilateral short fusion in patients less than 10 years old were performed. The aim is to preserve vertebral growth potential on the concavity, making for further correction of deformity as spine growth. As for unilateral fusion, previous studies had indicated it was effective and safe for very young children at long-term follow-up [16]. Excision of HV removes the primary cause of the scoliosis, which can achieve immediately good correction. However, for patients with congenital scoliosis caused by two ipsilateral HV more than 10 years old, the flexibility of curve is often lower and presenting more rigid in late adolescent than juvenile. Due to asymmetrical growth of hemivertebra, larger gap closure force after HV resection requires strong anchor points. Therefore, bilateral transpedicular screw fixation and fusion are indispensable for these patients.

The fusion level depends on severity of spine deformity, scope and flexibility of curve. For very young children, one vertebra above and below after HV resection were fixated and fused. If the scoliotic curve was large or with focal kyphosis, two vertebrae above and below after HV resection were fused. Of course, for severe and rigid scoliosis or kyphoscoliosis, more fusion level may be needed. Our results showed that correction rate of the major scoliotic and kyphotic curvatures were 74.2 and 65.3% respectively, which is similar to previously reported results for HV excision [4, 7, 10].

Coronal decompensation after HV resection with short fusion is also a problem which can’t be neglected. A large series reported by Li, et al. showed that the overall rate of coronal decompensation is approximately 10.1%, which including 179 cases in children younger than 5 years. Preoperative lower instrumented vertebra (LIV) translation and postoperative LIV disc angle were identified as two independent risk factors [17]. In our study, there are two cases presented the coronal decompensation. We think too short fusion segment and incomplete excision of proximal HV were main reasons. In young children with congenital scoliosis, fusion span determination relies mainly on optimal correction of scoliosis with solid screws and maximal preservation of spinal mobility and growth potential. However, more failure rates and residual curve progression were found in cases with double ispilateral full segmental HV if too short fusion or partial resection in corrective surgery. It is indicated that radical excision of HV could remove the causes of deformity immediately and stop the curve progressive.

Another concern is neurological complication after HV resection via posterior approach. Aydogan M, et al. [7] reported 11 cases with kyphoscoliosis due to HV by hemivertebrectomy and posterior instrumentation. None of the patients exhibited neurological problems associated with surgery. Our results suggest that HV excision in thoraco-lumbar region is not associated with an increased risk of neurological complications. Only in one patient undergoing one stage excision of three HV, intraoperative mild neurological injury was found. Based on our experience, the correction and balancing of congenital thoraco-lumbar curves are more effectively achieved by HV resection than other treatments. It should be undertaken only by those experienced with this technique.

Some cases presented residual curve progression after surgery, which may be attributed to a variety of factors, including multiple malformed vertebra, failure of segmentation, concave fused ribs, improper maneuver, shorter fusion level, incomplete HV resection and implants failure. Shi Z et al. investigate the causes of failure in the first operation and the revision procedure for patients with congenital scoliosis due to HV [18]. They suggested that limitations of the primary surgery, no or incomplete resection of HV, improper operation during surgery, improper internal fixation material and fixation scope were main cause of revision surgery. A study including 28 children less than 6 years old with HV were reported by Ruf M et al. [19]. They found two patients additional operations were performed because of new developing deformities. One was a bar formation at the operative site and an adjacent segment; another was a new bone mass at the site of the HV excision. They suggested that short fusion may increase the risk of a new deformity and may require re-operation, but this risk was acceptable to minimize the compromise of normal spinal development for very young patients. In our present study, three patients required additional surgery due to residual curve progression during follow-up. They had larger and rigid curve in coronal and sagittal plane before primary surgery. Incompletely excision of proximal HV was the major cause in two cases. Too short fusion and malformed vertebra growth was the major cause in another patient. The revision surgeries were performed and satisfactory outcome were achieved at the latest follow up.

In terms of anterior column reconstruction (ACR), a titanium mesh cage was used for anterior column support and fusion in patients who had residual anterior gap after HV resection. Aydogan M et al. [7] reported on 19 patients undergoing HV removal with 15 cases experiencing anterior mesh cage support to fill interbody space. They found anterior support in short segmental fusion could correct thoracolumbar kyphosis and increase the stability. In our cases, six cases showed anterior larger gap after HV excision; and titanium mesh cage were used in order to support anterior column and avoid spinal cord shorten too much. In addition to, it was beneficial to get solid fusion in bone cutting area.

This study has some limitations. First, it was a retrospective study with the inherent risk of data inaccuracy. Second, the wide range of the patients’ age makes this small series inhomogeneous and comparison is quite difficult. Finally, this study does not contain results about quality of life in the follow-up. Further study including quality of life and mental health status are needed in the future.

## Conclusion

Based on our experience, good correction and spinal balance can be achieved by one-stage posterior-only hemivertebra resection in patients with thoraco-lumbar kyphoscoliosis caused by two ipsilateral HV. Improvement of the segmental scoliosis and kyphosis curves is satisfactory and kept stable in long-term follow up. The complication of neurological injury is low but a technically demanding surgical procedure. More attention should be paid in residual curve progression after surgery.

## Data Availability

The datasets used and/or analyzed during the current study are available from the corresponding author upon reasonable request.

## Abbreviations

HV: Hemivertebra
CT: Computer tomography
MRI: Magnetic resonance imaging
TS: Trunk shift
SVA: Sagittal vertical axis
SPSS: Statistic Package for Social Science
AVT: Apex vertebra translation
VCR: Vertebra column resection
PSO: Pedicle subtraction osteotomy
LIV: Lower instrumented vertebra
ACR: Anterior column reconstruction

## References

[1] Mc Master MJ, Ohtsuka K (1982). The natural history of congenital scoliosis: a study of two hundred and fifty one patients. J Bone Joint Surg Am.

[2] McMaster MJ, Singh H (1999). Natural history of congenital kyphosis and kyphoscoliosis:a study of one hundred and twelve patients. J Bone Joint Surg Am.

[3] McMaster MJ, David CV (1986). Hemivertebra as a cause of scoliosis: a study of 104 patients. J Bone Joint Surg Br.

[4] Bollini G, Docquier PL, Viehweger E, Launay F, Jouve JL (2006). Thoracolumbar hemivertebrae resection by double approach in a single procedure: long-term follow-up. Spine (Phila Pa 1976).

[5] McMaster MJ (1998). Congenital scoliosis caused by a unilateral failure of vertebral segmentation with contralateral hemivertebrae. Spine (Phila Pa 1976).

[6] Lonstein JE, Winter RB, Moe JH, Bradford DS, Chou SN, Pinto WC (1980). Neurologic deficits secondary to spinal deformity. A review of the literature and report of 43 cases. Spine..

[7] Winter RB, Moe JH, Lonstein JE (1984). Posterior spinal arthrodesis for congenital scoliosis. An analysis of the cases of two hundred and ninety patients, five to nineteen years old. J Bone Joint Surg Am.

[8] Aydogan M, Ozturk C, Tezer M, Mirzanli C, Karatoprak O, Hamzaoglu A (2008). Posterior vertebrectomy in kyphosis, scoliosis and kyphoscoliosis due to hemivertebra. J Pediatr Orthop B.

[9] Ginsburg G, Mulconrey DS, Browdy J (2007). Transpedicular hemiepiphysiodesis and posterior instrumentation as a treatment for congenital scoliosis. J Pediatr Orthop.

[10] Crostelli M, Mazza O, Mariani M (2014). Posterior approach lumbar and thoracolumbar hemivertebra resection in congenital scoliosis in children under 10 years of age: results with 3 years mean follow up. Eur Spine J.

[11] Deviren V, Berven S, Smith JA, Emami A, Hu SS, Bradford DS (2001). Excision of hemivertebrae in the management of congenital scoliosis involving the thoracic and thoracolumbar spine. J Bone Joint Surg Br.

[12] Patel A, Ruparel S, Dusad T, Mehta G, Kundnani V (2018). Posterior-approach single-level apical spinal osteotomy in pediatric patients for severe rigid kyphoscoliosis: long-term clinical and radiological outcomes. J Neurosurg Pediatr.

[13] Zhuang Q, Zhang J, Li S, Wang S, Guo J, Qiu G (2016). One-stage posterior-only lumbosacral hemivertebra resection with short segmental fusion: a more than 2-year follow-up. Eur Spine J.

[14] Ruf M, Harms J (2002). Hemivertebra resection by a posterior approach: innovative operative technique and first results. Spine (Phila Pa 1976).

[15] Nakamura H, Matsuda H, Konishi S, Yamano Y (2002). Single-stage excision of hemivertebrae via the posterior approach alone for congenital spine deformity: follow-up period longer than ten years. Spine (Phila Pa 1976).

[16] Xue X, Zhao S (2018). Posterior hemivertebra resection with unilateral instrumented fusion in children less than 10 years old: preliminary results at minimum 5-year follow-up. J Orthop Surg Res.

[17] Li S, Chen ZH, Qiu Y, Xu L, Chen X, Du CZ (2018). Coronal Decompensation after posterior-only thoracolumbar Hemivertebra resection and short fusion in young children with congenital scoliosis. Spine (Phila Pa 1976).

[18] Shi Z, Li Q, Cai B, Yu B, Feng Y, Wu J, Li M, Ran B (2015). Causes of the failure and the revision methods for congenital scoliosis due to hemivertebra. Congenit Anom (Kyoto).

[19] Ruf M, Harms J (2003). Posterior hemivertebra resection with transpedicular instrumentation: early correction in children aged 1 to 6 years. Spine (Phila Pa 1976).

